# Comprehensive Strategies for Preventive Periodontal Care in Older Adults

**DOI:** 10.3390/geriatrics10030072

**Published:** 2025-05-25

**Authors:** Alice Kit Ying Chan, Yiu-Cheung Tsang, Stephanie Chu, Chun-Hung Chu

**Affiliations:** Faculty of Dentistry, The University of Hong Kong, Hong Kong 999077, China; dralice@hku.hk (A.K.Y.C.); elvis.yctsang@gmail.com (Y.-C.T.); stephchu.bds@gmail.com (S.C.)

**Keywords:** elderly, older adult, oral health, periodontal, plaque control, prevention

## Abstract

Background: Periodontal health is closely related to systemic health and crucial for healthy aging. Periodontal disease is prevalent among older adults due to declined systemic conditions, medication side effects, and reduced dexterity and cognition. Effective preventive care is essential to maintain periodontal health and promote oral and general health. Objective: The aim of this narrative review is to examine preventive periodontal care tailored for older individuals, with a focus on strategies to reduce the incidence of periodontal disease, maintain periodontal health, and improve the overall well-being of older adults. Findings: Preventive periodontal care includes mechanical plaque control, use of chemotherapeutic agents, lifestyle modifications, and regular professional periodontal care. Mechanical plaque control through regular toothbrushing and interdental cleaning remains the cornerstone of prevention. The use of adaptive aids and caregiver support is essential for maintaining the oral hygiene of older adults with physical limitations. Chemotherapeutic agents, such as chlorhexidine mouth rinses, can be used as adjunctive agents for plaque control. Lifestyle modifications, like smoking cessation and dietary adjustments, are crucial components of risk factor control. Professional periodontal care, including periodontal evaluation, risk factor control, tailored oral hygiene instruction, and professional mechanical plaque removal, are essential for the prevention and early detection and management of periodontal disease in older adults. Conclusions: This review underscores the importance of a multidisciplinary approach involving oral healthcare professionals, primary care providers, and caregivers to ensure patient-centered, integrated and comprehensive geriatric care to improve periodontal outcomes and overall well-being of older adults.

## 1. Introduction

The global demographic landscape is undergoing a significant transformation characterized by a substantial increase in the proportion of older adults. According to the United Nations, the number of people aged 65 or above is expected to double to approximately 1.6 billion by 2050, reaching one in six among the world population [1]. This demographic shift presents both opportunities and challenges for healthcare systems worldwide, particularly in the field of oral health, where the prevalence of periodontal disease among older adults is a growing concern [2,3]. Almost two-thirds of the global older adult population suffers from periodontal diseases [2]. One-third of German older adults aged 65 to 74 and one in seven Chinese older adults have severe periodontal diseases [4,5].

Periodontal disease encompasses a spectrum of conditions ranging from gingivitis, characterized by gingival bleeding and inflammation, to periodontitis, characterized by the destruction of periodontal ligament and alveolar bone [6]. Upon aging, the declined systemic conditions, medication side effects, and reduced dexterity and cognition exacerbate the susceptibility to periodontal disease [7]. The consequences of untreated periodontal disease extend beyond tooth loss and oral discomfort; emerging evidence links periodontal health to various systemic conditions [7,8]. 

The bidirectional relationship between periodontal health and diabetes mellitus has been well documented [9]. Recent evidence has proposed the association between periodontal disease and systemic conditions such as cardiovascular disease, rheumatoid arthritis and dementia through their shared inflammatory pathway in older adults [7,8]. Preventive periodontal therapy is, therefore, of paramount importance in aging societies to mitigate the risks associated with periodontal disease, enhance oral health, and promote general well-being among older individuals [2].

Periodontal disease is preventable and controllable. Oral health education and preventive care are the foundation for maintaining the periodontal health of older adults. Oral health education empowers older adults to understand the importance of oral health and its impact on general health [10]. Dental professionals play a pivotal role in disseminating this knowledge and providing tailored advice to address the unique challenges faced by older individuals, such as reduced dexterity and cognitive impairments. 

Oral health education also increases the dental awareness of older adults and motivates them to perform proper daily oral hygiene practices and seek timely professional oral care [11]. Preventive periodontal therapy aims to prevent periodontal diseases and manage gingival inflammation to avoid the progression into irreversible periodontitis [12]. It involves a comprehensive approach that integrates mechanical plaque control, the use of chemotherapeutic agents, lifestyle modifications, and regular professional care to prevent, early detect and manage periodontal disease [13]. 

The objective of this narrative review is to examine preventive periodontal care tailored for older individuals, with a focus on strategies to reduce the incidence of periodontal disease, maintain periodontal health, and improve the overall well-being of older adults.

## 2. Preventive Periodontal Care for Older Adults

This review was based on English publications, including reviews and clinical studies identified in PubMed and Google Scholar up to March 2025 using the keywords “plaque control”, “periodontal disease”, “periodontal health”, “gingival health”, “periodontal care”, “preventive oral care”, “aged”, “older adults”, and “elderly”. 

**Figure 1:**
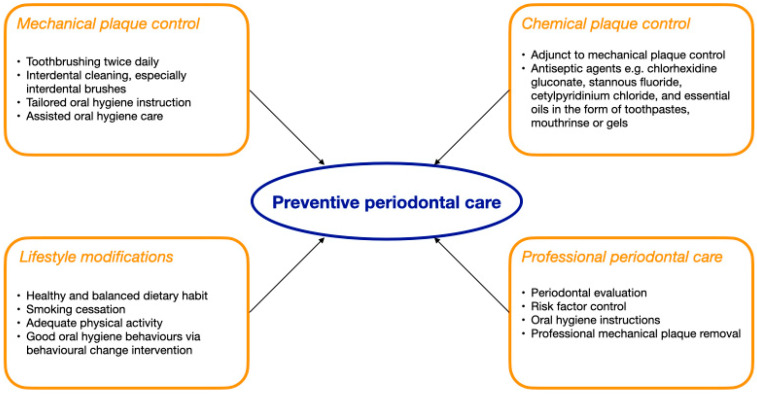
Strategies of preventive periodontal care for older adults.

Four strategies of preventive periodontal care, including mechanical plaque control, use of chemotherapeutic agents, lifestyle modifications, and regular professional periodontal care, were identified. Figure 1 presents the four strategies of preventive periodontal care for older adults.

### 2.1. Mechanical Plaque Control

Dental biofilm, also called dental plaque, is the prime causative agent of periodontal disease [14]. Mechanical plaque control is the key to preventing periodontal disease and maintaining periodontal health. Physiological changes and chronic medical conditions upon aging can impact an individual’s ability to maintain optimal oral hygiene and seek professional oral care [7]. These changes include reduced manual dexterity and functional ability due to arthritis, impaired vision, and declined cognition [7]. Traditional mechanical plaque control, such as manual toothbrushing and flossing, becomes more challenging for older adults. Therefore, adapting mechanical plaque control strategies to meet the unique needs of this age group is essential. Various oral hygiene tools, tailored oral hygiene instruction, assisted oral hygiene care, and professional mechanical plaque control may address the challenges faced by older adults during oral hygiene practice.

#### 2.1.1. Toothbrushing

The toothbrush is the primary tool for mechanical plaque control and is essential in cleaning facial and lingual/palatal tooth surfaces [15]. All people should brush their teeth twice daily for at least 2 min to prevent periodontal disease [14]. A study found that older adults using the Bass toothbrushing method, in which bristles were aligned 45 degrees to the gingival margin, had more reduction in dental plaque than those using other methods [16]. Although toothbrushing seems to be an easy habitual routine, toothbrushing can be challenging for older adults who have physical and cognitive difficulties [7]. Adaptive aids for manual toothbrushes are available in the market to enhance mechanical plaque control for older adults. For example, toothbrush grips can be attached to the handle of a toothbrush to make it easier to hold. A toothbrush with extended handles can provide better access to the oral cavity and improve the user experience. These aids can help older adults maintain independence in their oral hygiene practices. In addition to manual toothbrushes, electric toothbrushes, which require less manual dexterity and effort, are designed to simplify and facilitate toothbrushing practice. Electric toothbrushes have been recommended for older adults who are with a physical disability or cognitive decline [17]. However, comparative studies found both manual and electric toothbrushes effective in improving plaque control in older adults, and no superiority between these two types of toothbrushes has been detected [18,19].

#### 2.1.2. Interdental Cleaning

Interdental cleaning together with toothbrushing can remove plaque in interdental areas and improve periodontal health more than toothbrushing alone [15]. Dental floss, interdental brushes, and water floss are available for interdental cleaning. Flossing is the most popular interdental cleaning method promoted in commercial or oral health education materials. However, flossing requires delicate skills and fine dexterity and can be difficult for older adults, especially those with limited hand strength or coordination. In the 11th European Workshop in Periodontology, a consensus by Chapple et al. listed interdental brushes as the first choice for interproximal plaque removal except at the interdental area that cannot be passed by the interdental brushes without trauma [14]. A randomized clinical trial detected more improvement in clinical periodontal outcomes in terms of plaque accumulation and probing depth in patients using an interdental brush than those using dental floss [20]. Older adults usually have enlarged interdental space resulting from cumulative periodontal disease, and will be more beneficial in using interdental brushes. Water floss, which uses a stream of pulsating water to clean between teeth and along the gum line, can be an alternative for older adults with reduced dexterity due to its ease of use [21]. When used as an adjunct to toothbrushing, water floss was more effective in removing plaque from all surfaces than waxed dental floss [22].

#### 2.1.3. Tailored Oral Hygiene Instruction

Tailored oral hygiene instruction is important because it addresses the unique needs and conditions of each individual, ensuring that the advice provided is relevant and effective. By customizing instructions based on factors such as age, dental history, and specific oral health issues, patients are more likely to follow the recommendations and achieve better outcomes. This personalized approach helps improve overall oral hygiene, prevents dental diseases, and promotes long-term oral health [13]. Oral healthcare professionals play the main role in educating older adults or their caregivers on proper toothbrushing and interdental cleaning techniques. Customized instructions and hands-on demonstrations can help individuals develop the skills needed to maintain good oral hygiene. Due to the recent development of digital technology, the use of mobile devices in the form of videotaping oral hygiene instruction may also enhance oral hygiene practice [23]. Older adults who may be forgetful can replay the video to remember the related advice and techniques during their oral hygiene practice [24]. Older adults can use disclosing tablets to evaluate and monitor their self-performed oral hygiene practice.

#### 2.1.4. Assisted Oral Hygiene Care

Older adults with reduced manual dexterity or impaired cognition may not be able to properly perform oral hygiene practice and become more dependent on self-care activities [10]. Caregivers bear the responsibility of supervising or providing oral hygiene care for dependent older adults [25]. Toothbrushing is a well-learned habitual skill and can be maintained or improved with the support of caregivers through reminders or cueing [26]. However, a lack of knowledge and the required resources are the main barriers for caregivers in providing assisted oral hygiene care [27]. Caregivers may not know the proper technique and the available oral hygiene aids, e.g., extended handles for manual toothbrushes, electric toothbrushes or water flossing to assist oral hygiene practice for older adults. They may not know how to evaluate their performed assisted oral hygiene practice. A recent clinical trial proved that, with the proper training and support to home caregivers, e.g., individualized demonstration of oral hygiene practice and provision of electric toothbrushes and disclosing tablets, community-dwelling older adults with cognitive impairment showed an improvement in plaque control and gingival condition from the assisted oral hygiene care [27]. Assisted oral hygiene care is crucial for individuals who are unable to perform oral hygiene tasks independently, such as the elderly, disabled, or those with limited dexterity. Providing assistance ensures that these individuals maintain proper oral health, preventing dental diseases and improving their overall quality of life.

#### 2.1.5. Professional Mechanical Plaque Control

Regular dental visits for professional mechanical plaque removal can facilitate the daily oral hygiene practice. During professional mechanical plaque removal, dental calculus, which is plaque retentive and is formed from dental plaque missed during daily oral hygiene care, will be removed [12]. This professional preventive care can ensure the efficiency of daily oral hygiene practice and aid in maintaining the periodontal health of older adults.

Mechanical plaque control is a cornerstone of preventive periodontal care for older adults. Electric toothbrushes, interdental brushes, water floss, and adaptive aids can enhance the accessibility of plaque control. Dental professionals provide tailored oral hygiene instruction, and professional mechanical plaque removal can further improve the effectiveness of mechanical plaque control in older adults. Trained caregivers provide assisted oral hygiene care for dependent older adults. By addressing their specific needs, older adults can perform efficient mechanical plaque control to improve their oral health and overall well-being.

### 2.2. Use of Chemotherapeutic Agents

Mechanical plaque control is the mainstream method for preventing periodontal disease and maintaining oral health; however, it relies on patient motivation, cooperation, and manual dexterity. Chemical plaque control using antiseptic substances such as chlorhexidine gluconate, stannous fluoride, cetylpyridinium chloride and essential oils serve as valuable adjuncts for older adults with cognitive and functional difficulties to assist plaque control [14]. Their adjunctive use has also been found beneficial for the management of gingival inflammation [14,28].

#### 2.2.1. Chlorhexidine Gluconate

Chlorhexidine gluconate is used to minimize microbial load in the oral cavity, prevent dental plaque accumulation, and reduce gingival inflammation [29]. Chlorhexidine works by disrupting the bacterial cell membrane, which leads to cell death. It has a substantivity of up to 12 h to provide prolonged antimicrobial activity [29]. Long-term use of chlorhexidine can lead to side effects such as tooth staining, altered taste sensation, and mucosal irritation [29]. Therefore, its use is indicated in occasions such as acute periodontal infection or when oral hygiene practice is not feasible. Chlorhexidine is most commonly available as a mouth rinse, which is convenient and easy to use and reaches all niches of the oral cavity simultaneously. There is high-quality evidence to support the use of chlorhexidine mouth rinse as an adjunct to mechanical plaque control in reducing plaque accumulation and gingival inflammation in up to 6 months [30]. As the plaque inhibition by chlorhexidine mouth rinse appears to be dose-dependent, similar effects from high-concentration chlorhexidine mouth rinse can be achieved with higher volumes of low-concentration chlorhexidine mouth rinse [31]. For older adults with a reduced gag reflex and increased risk of aspiration, chlorhexidine gel can be applied along the gingival margins locally.

#### 2.2.2. Stannous Fluoride

Stannous fluoride has been used since the 1940s and is available in gel, toothpaste and mouth rinse. Stannous fluoride has strong antimicrobial properties, which help reduce bacteria that cause plaque, gingivitis, and bad breath. This makes it particularly effective in promoting overall oral health beyond just cavity prevention. It interferes with bacterial metabolism, reduces bacterial adhesion and cohesion and affects the acid production by bacteria [32]. Stannous fluoride has been shown to have anti-inflammatory effects that can help reduce gum inflammation and bleeding, making it beneficial for managing gingivitis [32]. An astringent taste and dental staining are the main side effects of stannous fluoride. A systematic review reported reduced levels of plaque accumulation and gingival inflammation after using stannous fluoride formulations [32]. There is weak evidence supporting the use of stannous fluoride in the management of gingivitis [33].

#### 2.2.3. Cetylpyridinium Chloride

Cetylpyridinium chloride is a monocationic quaternary ammonium compound [32]. It is commonly used in mouth rinse and oral care products for its antimicrobial properties. It disrupts the cell membrane of bacteria, leading to the loss of cellular components and alteration of cellular metabolism [32]. Cetylpyridinium chloride is effective in killing a wide range of bacteria, including those responsible for plaque formation, gingivitis, and bad breath (halitosis). Its ability to reduce the bacterial load in the mouth makes it a valuable ingredient in oral care products. Its side effects are similar to those of chlorhexidine but with less frequency and intensity [32]. Cetylpyridinium chloride is generally well-tolerated, with a good safety profile for use in over-the-counter oral care products. A systematic review reported a small but significant additional benefit from cetlylpyridinium chloride containing mouthrinse in reducing plaque accumulation and gingival inflammation as an adjunct to toothbrushing [34].

#### 2.2.4. Essential Oils

Essential oil-based mouthrinse, such as those containing thymol, eucalyptol, and menthol, offers an alternative to chlorhexidine mouth rinse [32]. Essential oils disrupt the bacterial cell wall and inhibit enzyme activity [32]. Studies showed that essential oil-based mouthrinse reduced plaque accumulation and gingival inflammation but to a lesser degree than chlorhexidine mouth rinse [35]. While less potent than chlorhexidine, essential oil-based mouth rinse is suitable as an alternative for older adults who need long-term control of gingival inflammation because of the fewer side effects [35] Table 1 summarizes the common chemotherapeutic agents for chemical plaque control. Chemotherapeutic agents play a vital role in the prevention and management of periodontal disease in older adults. With careful consideration of the potential benefits and side effects, these agents can enhance the effectiveness of mechanical plaque control and maintain the periodontal health of older adults.

### 2.3. Lifestyle Modifications

Several health behaviors, such as dietary habits, smoking, physical activity and oral hygiene habits, are associated with periodontal health [13]. Promoting lifestyle modifications for the prevention of oral disease involves educating individuals on the importance of a balanced diet, maintaining good physical activities, and proper oral hygiene practices [13]. Additionally, encouraging the reduction of harmful habits like smoking can significantly improve oral health [36]. Modifying these behavioral risk factors can reduce the risk of periodontal disease and aid in controlling the progression.

#### 2.3.1. Dietary Habit

Emerging evidence indicated that periodontal health is associated with dietary habits in older adults [37]. A diet rich in carbohydrates and saturated fats may trigger the immune-mediated inflammatory response and increase the risk of periodontal disease [38], whereas a diet rich in polyunsaturated fatty acids, fibers, and antioxidant micronutrients may retard the progression of periodontal disease in older adults [39]. Energy requirement decreases with age, and older adults may have a reduced dietary intake and hence be at risk of micronutrient deficiency. Edentulism, discomfort from existing prostheses, or systemic conditions such as dementia and sarcopenia may reduce the masticatory efficiency of older adults and impact the food intake [40,41,42]. Nutritional deficiency or imbalance not only inversely affects the periodontal health but also the overall well-being of older adults. Oral healthcare professionals should conduct dietary analysis and provide advice to ensure older adults, particularly those who are frail and dependent, adopt healthy, well-balanced eating habits based on the food pyramid to maintain periodontal health and overall well-being [37]. Moreover, oral healthcare professionals should arrange referrals to nutritionists if they suspect older adults are at risk of nutritional deficiency or imbalance [37].

#### 2.3.2. Smoking

Smoking is a well-established risk factor for periodontal disease [43]. Smoking cessation is beneficial in hampering the progression of periodontal health and reducing the risk of periodontal-associated tooth loss [43]. The World Health Organization stated that oral healthcare professionals have the greatest potential to promote smoking cessation in the primary healthcare system because they are able to approach a large pool of the population and provide individualized feedback based on their oral condition affected by smoking [44]. A US report showed that a 3 min brief intervention on smoking habits by oral healthcare professionals can increase the smoking cessation rate by 30% [45]. Oral healthcare professionals can promote smoking cessation to older adults by arranging behavioral counseling, using nicotine replacement therapies, implementing the 5A model (ask, advise, assess, assist, and arrange) and referring them to smoking cessation programs when indicated [46].

#### 2.3.3. Physical Activity

The World Health Organization has listed physical activity as one of the four modifiable behavioral risk factors for the prevention of non-communicable diseases [47]. The impact of physical activity on periodontal health has recently gained more attention. Cross-sectional studies reported an association between increased physical activity and improved periodontal health in terms of clinical attachment loss, probing depths and bleeding on probing [48]. The proposed mechanism is that physical activity may reduce inflammatory biomarkers and, hence, systemic inflammation. It may also modify the shared risk factors such as diabetes mellitus and obesity [48]. Therefore, exercise interventions such as low-intensity regular walking may be beneficial for the prevention and management of periodontal disease in older adults [48,49].

#### 2.3.4. Oral Hygiene Habits

Daily oral hygiene practice is an essential lifestyle behavior to remove dental plaque and maintain periodontal health. Its long-term success in improving oral health depends on patient compliance with behavior changes [12]. When compared with other age groups, older adults find it difficult to comply with oral hygiene practices. They may forget to brush their teeth or use the correct toothbrushing method due to the declined cognitive function. Their underlying medical conditions or related medication may reduce their motivation in oral hygiene practice [50]. They may have difficulties in buying and organizing oral hygiene tools because of their reduced physical and cognitive function. Behavioral change intervention is needed to enhance their oral hygiene practice [12].

##### 2.3.4.1. The 5S Methodology

The 5S methodology has been recommended for integration into oral hygiene practice to enhance the behavioral change of plaque control in older adults [51]. The 5S methodology was originally developed for improving efficiency and organization in the workplace [52]. It consists of five steps: sort, set in order, shine, standardize, and sustain. Table 2 summarizes the five steps of the proposed 5S integrated behavioral change oral hygiene intervention for older adults. The 5S integrated behavioral change oral hygiene intervention will create a clean and well-organized working environment to ensure safety, an important component in geriatric care for older adults. With all the oral hygiene tools set in order, it can ease the execution difficulties for older adults. The standardized repeated oral hygiene routine can enhance memory and compensate for age-related cognitive decline in older adults. All steps help increase older adults’ compliance with oral hygiene practices and improve periodontal health in the long term. Integrating the 5S methodology into oral hygiene practice not only helps in maintaining periodontal health but also fosters a sense of independence and self-efficacy among older adults.

In addition, periodontal disease shares some common risk factors with other non-communicable diseases, such as diabetes and cardiovascular diseases [53]. Addressing these behavioral risk factors not only improves the periodontal health but also the systemic health and overall well-being of older adults.

### 2.4. Regular Professional Periodontal Care

The *White Paper on Prevention and Management of Periodontal Diseases for Oral Health and General Health* by the World Health Organization highlighted the importance of regular professional periodontal care for the prevention, early detection and management of periodontal diseases [54]. Table 3 shows the four components of professional periodontal care. Each dental visit should cover all four components for preventive periodontal care. Evidence found little value in having professional mechanical plaque removal without oral hygiene instruction in reducing gingival inflammation [12]. Repeated and tailored oral hygiene instruction is the key element for success in maintaining periodontal health [12]. Older adults are more susceptible to periodontal diseases [7]. Aging can lead to changes in the oral cavity, such as reduced salivary flow rate and chronic medical conditions like diabetes, cardiovascular diseases and dementia [7]. All these changes can exacerbate periodontal problems [7]. Oral healthcare professionals should assess each older adult’s periodontal risk based on the level of infection (full mouth bleeding scores), periodontal-related tooth loss, systemic conditions, environmental and behavioral factors such as smoking and oral hygiene behaviors, and level of dependency to customize the frequency and content of each professional periodontal care visit [55,56]. Oral healthcare professionals should address all the risk factors in each visit to prevent or retard the periodontal diseases in older adults [54].

As regular professional intervention is crucial in the prevention of periodontal disease, it is important to ensure the dental attendance of older adults through oral health education. Primary healthcare professionals should emphasize the link between oral and general health to older adults and encourage them to have regular dental visits.

## 3. Public Health Initiatives for Preventive Periodontal Care

Physical disability and financial difficulties are the two main barriers to seeking regular preventive periodontal care for older adults and hence deter timely diagnosis and management of periodontal disease in older adults [57]. Removal of these barriers relied on national oral healthcare policies. Physical access can be facilitated through transportation services and mobile dental clinics for older adults [58]. Teledentistry has recently been utilized to provide oral healthcare to vulnerable groups or areas with a shortage of oral healthcare services [59]. The application of teledentistry in oral diagnosis and oral health education has shown promising results and can be considered for older adults who have physical difficulties [60].

Dental treatment has been subsidized in various forms in many countries for those in need [61,62]. Government-subsidized dental care service programs, non-government organizations, and dental schools may offer reduced-cost or free dental services for older adults [61,62]. Dental professionals, healthcare professionals, or social workers can help provide related information or schedule appointments with those organizations for older adults with financial difficulties to ensure they receive regular dental care.

Periodontal health is closely related to systemic health and healthy aging [53]. Preventive periodontal care should be integrated into broader public health initiatives to promote the overall well-being of older adults [63]. Policymakers must recognize the significance of periodontal health in healthy aging and allocate appropriate resources to support community-based preventive periodontal care programs [63]. Geriatric oral healthcare should be integrated into primary healthcare services [46]. Older adults should receive patient-centered, integrated and comprehensive care through an interdisciplinary collaboration between oral and other healthcare providers such as geriatricians, primary healthcare professionals, dietitians, physiotherapists, and social workers to enhance oral and general health [64]. Collaborative care models can facilitate timely referrals, coordinate care plans, utilize a common risk factor control approach and provide holistic management of periodontal diseases in older adults [46,64]. This approach can also ensure that periodontal health and overall oral health are not treated in isolation but as an integral part of overall health management.

## 4. Conclusions

Periodontal health is closely related to systemic health and healthy aging. The importance of preventive periodontal care in aging societies cannot be overlooked. Mechanical plaque control, use of chemotherapeutic agents, lifestyle modifications, and regular professional periodontal care are paramount for maintaining periodontal health in older adults. A multidisciplinary approach involving oral healthcare professionals, primary healthcare providers, and caregivers can ensure patient-centered, integrated, and comprehensive care to improve periodontal outcomes and the overall well-being of older adults.

## Figures and Tables

**Table 1:** Chemotherapeutic agents for chemical plaque control.

Active Agent	Antimicrobial Mechanisms	Delivery Format	Side Effects
Chlorhexidine gluconate	▪ To disrupt the cell membrane	▪ Mouthrinse ▪ Toothpaste ▪ Gel	▪ Tooth staining, ▪ Taste alteration, ▪ Mucosal irritation
Stannous fluoride	▪ To interfere with metabolism ▪ To reduce adhesion and cohesion ▪ To affect the acid production	▪ Mouthrinse ▪ Toothpaste ▪ Gel	▪ Tooth staining, ▪ Astringent taste
Cetylpyridinium chloride	▪ To disrupt the cell membrane ▪ To alter metabolism	▪ Mouthrinse	▪ Tooth staining, ▪ Taste alteration, ▪ Mucosal irritation
Essential oils	▪ To disrupt cell wall ▪ To inhibit enzyme activit	▪ Mouthrinse	-

**Table 2:** The five steps of the 5S-integrated behavioral change oral hygiene intervention.

Step		Objective	Scopes of Work
**1**	**Sort**	To Reduce confusion	▪ Declutter oral hygiene workplace ▪ Label and organize the contents of containers
**2**	**Set in order**	To Keep tools more accessible	▪ Organize all oral hygiene tools at designated spots in order following the practice sequence
**3**	**Shine**	To Ensure hygiene and safety	▪ Clean and fix the oral hygiene workplace ▪ Clean or replace the oral hygiene tools ▪ Ensure the working environment is clean and safe, e.g., adequate lighting and absence of dangers
**4**	**Standardize**	To Enhance adherence	▪ Set up a regular oral hygiene practice schedule ▪ Standardize oral hygiene methods ▪ Maintain the setup of oral hygiene in the workplace
**5**	**Sustain**	To Maintain long-term oral behavior change	▪ Evaluate patient adherence regularly ▪ Adjust the routine and setting of oral hygiene practice to further enhance adherenc

**Table 3:** Four components of professional periodontal care.

Objectives	Scopes of Work
**Periodontal Evaluation**	
▪ To evaluate the effectiveness of daily plaque control ▪ To early detect periodontal disease	▪ Assess oral hygiene status ▪ Assess periodontal conditions
**Risk factor control**	
▪ To detect and control periodontal risk factors	▪ TPerform the risk assessment ▪ Advocate lifestyle modification ▪ Collaborate with primary healthcare workers
**Oral Hygiene Instructions**	
▪ To enhance patient adherence and daily plaque control	▪ Utilize behavioral change intervention ▪ Provide personalized oral hygiene instructions
**Professional mechanical plaque removal**	
▪ To facilitate daily plaque control	▪ Remove plaque and calculus ▪ Polish tooth surfaces
